# Clinical and Functional Characteristics of Patients with Unclassifiable Interstitial Lung Disease (uILD): Long-Term Follow-Up Data from European IPF Registry (eurIPFreg)

**DOI:** 10.3390/jcm9082499

**Published:** 2020-08-03

**Authors:** Ekaterina Krauss, Mustapha El-Guelai, Joern Pons-Kuehnemann, Ruth C. Dartsch, Silke Tello, Martina Korfei, Poornima Mahavadi, Andreas Breithecker, Ludger Fink, Mark Stoehr, Raphael W. Majeed, Werner Seeger, Bruno Crestani, Andreas Guenther

**Affiliations:** 1 European IPF Registry & Biobank (eurIPFreg/bank), 35392 Giessen, Germany; ekaterina.krauss@innere.med.uni-giessen.de (E.K.); mustapha.el-guelai@innere.med.uni-giessen.de (M.E.-G.); ruth.c.dartsch@innere.med.uni-giessen.de (R.C.D.); silke.tello@innere.med.uni-giessen.de (S.T.); martina.korfei@innere.med.uni-giessen.de (M.K.); poornima.mahavadi@innere.med.uni-giessen.de (P.M.); mark.stoehr@innere.med.uni-giessen.de (M.S.); raphael.majeed@chiru.med.uni-giessen.de (R.W.M.); werner.seeger@innere.med.uni-giessen.de (W.S.); bruno.crestani@aphp.fr (B.C.); 2 Universities of Giessen and Marburg Lung Center (UGMLC), Member of the German Center for Lung Research (DZL), 35392 Giessen, Germany; andreas.breithecker@radiol.uni-giessen.de (A.B.); fink@patho-uegp.de (L.F.); 3 Medical Statistics, Institute of Medical Informatics, Justus-Liebig University of Giessen; 35392 Giessen, Germany; joern.pons@informatik.med.uni-giessen.de; 4 Gesundheitszentrum Wetterau, 61231 Bad Nauheim, Germany; 5 Institute of Pathology, Cytology, and Molecular Pathology, 35578 Wetzlar, Germany; 6 Cardio-Pulmonary Institute (CPI) 35392 Giessen, Germany; 7 Institute National de la Sainté et de la Recherche Médicale, Hopital Bichat, Service de Pneumologie, 75018 Paris, France; 8 Agaplesion Lung Clinic Waldhof-Elgershausen, 35753 Greifenstein, Germany

**Keywords:** unclassifiable interstitial lung disease (uILD), idiopathic pulmonary fibrosis (IPF), European Registry for idiopathic pulmonary fibrosis (eurIPFreg), interstitial lung diseases (ILD), Health-related quality of life (HRQoL)

## Abstract

(1) Aim of the study: In spite of extensive research, up to 20% of interstitial lung diseases (ILD) patients cannot be safely classified. We analyzed clinical features, progression factors, and outcomes of unclassifiable ILD (uILD). (2) Methods: A total of 140 uILD subjects from the University of Giessen and Marburg Lung Center (UGMLC) were recruited between 11/2009 and 01/2019 into the European Registry for idiopathic pulmonary fibrosis (eurIPFreg) and followed until 01/2020. The diagnosis of uILD was applied only when a conclusive diagnosis could not be reached with certainty. (3) Results: In 46.4% of the patients, the uILD diagnosis was due to conflicting clinical, radiological, and pathological data. By applying the diagnostic criteria of usual interstitial pneumonia (UIP) based on computed tomography (CT), published by the Fleischner Society, 22.2% of the patients displayed a typical UIP pattern. We also showed that forced vital capacity (FVC) at baseline (*p* = 0.008), annual FVC decline ≥10% (*p* < 0.0001), smoking (*p* = 0.033), and a diffusing capacity of the lung for carbon monoxide (DLco) ≤55% of predicted value at baseline (*p* < 0.0001) were significantly associated with progressive disease. (4) Conclusions: The most important prognostic factors in uILD are baseline level and decline in lung function and smoking. The use of Fleischner diagnostic criteria allows further differentiation and accurate diagnosis.

## 1. Introduction

Interstitial lung diseases (ILD) are a heterogeneous group of parenchymal fibrosing lung diseases characterized by varying degrees of inflammation and scarring [1]. Some of the ILD entities are additionally associated with a progressive fibrosing phenotype, being clinically characterized by progressive respiratory symptoms, a faster decline of lung function, limited response to antifibrotic or immunomodulatory therapies, decreased health-related quality of life (HRQoL), and as a consequence, early death [2,3].

ILDs may occur due to a known cause such as drugs, connective tissue disease (CTD), hypersensitivity to inhaled organic antigens (hypersensitivity pneumonitis, HP), or sarcoidosis; whilst others, especially sporadic idiopathic interstitial pneumonias (IIPs), have no identifiable origin [4]. Of them, idiopathic pulmonary fibrosis (IPF) is associated with the highest burden and poor prognosis [5,6,7]. Apart from IPF, other IIPs include idiopathic non-specific interstitial pneumonia (NSIP), respiratory bronchiolitis-ILD (RB-ILD), desquamative interstitial pneumonia (DIP), cryptogenic organizing pneumonia (COP), acute interstitial pneumonia (AIP), and some other rare forms [8,9].

The differential diagnosis of ILD can be challenging, and it requires detailed consideration of clinical, radiological, and histopathological features by a multidisciplinary team (MDT) [2]. The MDT discussion has become the new gold standard to diagnose and manage ILDs, enhancing the correctness of the diagnosis [10]. Nevertheless, in up to 20% of ILD patients, a specific diagnosis cannot be defined, even after thorough MDT discussion. In such a situation, the disease would be labeled as an “unclassifiable ILD” (uILD) [11]. This term was introduced in the American Thoracic Society/European Respiratory Society (ATS/ERS) Consensus Classification of the IIPs in 2002, as a description of an ILD subgroup that (at least temporarily) cannot be classified within the confines of the current diagnostic framework [12]. A recent International Working Group suggested that uILD should be defined by the absence of a leading diagnosis that is considered more likely than not (after MDT discussion of all available information) [13].

An ILD might be labeled unclassifiable for various reasons. As suggested by Hyldgaard et al., in most cases, the inability to certainly classify ILD is the result or combination of the following aspects: (1) a biopsy was not performed (and/or clinical and radiological data were insufficient for accurate diagnosis); (2) histopathological results were inconsistent or overlapping; (3) discrepancy between clinical, radiological, and pathological findings; and (4) significant overlap between clinical and/or radiological features [11]. Common histological patterns of uILD are often overlapping, including e.g., usual interstitial pneumonia (UIP) with bronchiolo-centric fibrosis, NSIP with organizing pneumonia, UIP with NSIP-like changes, and UIP with diffuse alveolar damage [14,15]. Further studied factors for diagnostic assessment are a fibrotic score and the percentage of fibroblastic foci [16].

Another entity currently included in the category of uILD is interstitial pneumonia with autoimmune features (IPAF) [17]. Even though a variety of clinical, serological, or pulmonary morphological autoimmune factors may be recognized, individuals with IPAF do not have a defined connective tissue disease (CTD), based on established rheumatological criteria [18]. As shown by Fischer et al., an estimated 7% of patients with uILD might be diagnosed with IPAF; these patients cannot be diagnosed with a specific type of IIP or CTD-ILD, but they appear to have similar or better survival rates than patients with IPF [19].

A study performed by Cottin et al. found out that some patients with ILD may develop a progressive-fibrosing phenotype, which is associated with worsening respiratory symptoms, lung function decline, limited response to immunomodulatory therapies, decreased HRQoL, and potentially, early death [2]. Knowingly, the mortality in chronic ILD can be predicted using the Gender, Age, Physiology (GAP) score, which is a clinical predictor model based on sex, age, and pulmonary function [20].

As a result of progress made in the field of ILD aetiology and pathogenesis, and due to improvements in radiological diagnostics, the existing ATS/ERS/JRS/ALAT guideline was updated in 2015 [21]. Still, in spite of the research over the last years, uILD still remained a disease without causal therapy regimes, and as a consequence, high unmet medical need. As this group is quite heterogeneous and the response to the therapy shows great diversity, it is essential to identify reliable prognostic factors indicating the risk of decline in pulmonary function test (PFT) and treatment response in a comprehensive, non-selected patient’s cohort.

The aims of this study were to describe the detailed clinical characteristic of the Giessen uILD cohort included in the European IPF Registry (eurIPFreg), as well as to define the prognostic factors of PFT decline, impact on HRQoL, and survival analysis.

## 2. Materials and Methods

### 2.1. Data Collection

In this study, a total of 140 uILD patients from the European IPF register (eurIPFreg), sites “Universities of Giessen and Marburg Lung Center” (including Agaplesion Lung Clinic Waldhof-Elgershausen) have been analyzed on the basis of clinical and PFT data, covering the period from November 2009 until January 2019. The cohort was followed up until January 2020. The disease progression was defined as change in annual rate of forced vital capacity (FVC) decline of 10 percentage points of the predicted value (% pred.).

The eurIPFreg is an Internet-based, multicenter registry with the intention to better characterize ILD within the framework of translational research [5]. The eurIPFreg received positive votes from the Ethics Committee of Justus-Liebig-University of Giessen (Nr.111/08) and is listed in ClinicalTrials.gov (NCT02951416). Patients above 18 years old with ILD who signed the informed consent were enrolled into the eurIPFreg and thus selected for this study. The research was conducted strictly according to the principles of the Declaration of Helsinki.

The clinical data were collected at the time of enrollment (baseline) and in 3 to 12 months intervals via patient and physician questionnaires [5]. There were no specific exclusion criteria, although patients with an absence of signed informed consent as well as pregnancy were not enrolled into the eurIPFreg. Each patient’s diagnosis was evaluated on the basis of the respective ATS/ERS/JRS/ALAT guideline [21,22,23,24]. The diagnosis of uILD was applied only when all findings suggested no alternative ILD diagnosis in a MDT discussion including at least one pulmonologist, pathologist, and radiologist with experience in ILD. For the MDT discussion, the patients’ data included all ILD relevant clinical and serological information, high-resolution computed tomography (HRCT) scans, together with pathological specimens (if available).

In case there were observations suggestive of a rheumatoid disease (such as pathological findings in an auto-antibody screen, or in the physical examination or hints in patient’s history), the patient was seen by a rheumatologist, and this information was included in the MDT discussion.

The whole uILD cohort is a subject of continuous verification of the certainty in uILD diagnosis; from the initial uILD cohort (*n* = 152), 12 patients were over time re-classified as IPF, and have therefore been excluded from this study.

HRQoL was measured at baseline using the Short Form Health Survey (SF-36). The aim of SF-36 is to characterize and understand the relationship between the selected baseline clinical covariates, the physical component score (PCS), and the mental component score (MCS) [25]. This questionnaire is a validated instrument for assessing HRQoL and has been applied to a variety of chronic medical conditions, including IPF [7]. The SF-36, as described in the name, is a 36-item patient-reported questionnaire that evaluates eight health domains: physical functioning (10 items), bodily pain (2 items), role limitations due to physical health problems (4 items), role limitations due to personal or emotional problems (4 items), emotional well-being (5 items), social functioning (2 items), energy/fatigue (4 items), and general health perceptions (5 items). Scores for each domain range from 0 to 100, with a higher score defining a more favorable health state [7].

### 2.2. Quality of Data and Statistical Analysis

The quality of data in eurIPFreg was controlled by internal plausibility checks, in which different items were put into a logical context, causing the generation of queries in case inconsistent entries were noted (e.g., if physician’s and patient´s report were not consistent with regard to signs of underlying collagen/vascular disease).

All statistical procedures were performed using SPSS 24 (SPSS, IBM Corp). For baseline data, the summary descriptive statistic was generated with categorical data displayed as absolute numbers and relative frequencies. Continuous data were shown as mean values (± standard deviation (SD)) for normally distributed data or as median values (± interquartile range) for nonparametric data. Comparisons between groups were performed using t-test, Chi² test, or Mann–Whitney U test, as appropriate.

The evaluation of the risk factors for PFT decline and mortality was performed by Cox regression analyses with covariates. Survival analyses were calculated in dependency on:

- Age (pts. younger and older than 60 years)
- Annual decline in FVC in % pred.
- Smoking history
- Gender–Age–Physiology Index (GAP) at baseline (GAP stages I, II, and III). GAP score is a clinical predictor model of disease progression and mortality in ILD based on sex, age, and pulmonary function [20].
- Diffusing capacity of the lung for carbon monoxide (DLco) in % pred. at baseline
- Six minutes walking distance (6MWD) in meters at baseline
- UIP pattern in HRCT at the timepoint of a diagnosis; additionally, the whole cohort was retrospectively evaluated, applying Fleischner Society criteria [21].

The outcome data were presented by Kaplan–Meier curves.

## 3. Results

### 3.1. Demographics and Descriptive Characteristic of uILD Cohort

As per January 2019, a total of 1634 patients (including non-ILD controls) were enrolled into the eurIPFreg UKGM sites Giessen and Greifenstein; of them, 920 were ILD patients. The ILD cohort included 470 IIP patients, of whom 271 were IPF subjects and 140 were patients with uILD (15.2% of a total ILD cohort, 26.4% of an IIP cohort). The descriptive characteristic of the uILD cohort at baseline is outlined in Table 1.

### 3.2. Comorbidities in uILD

**Figure 1:**
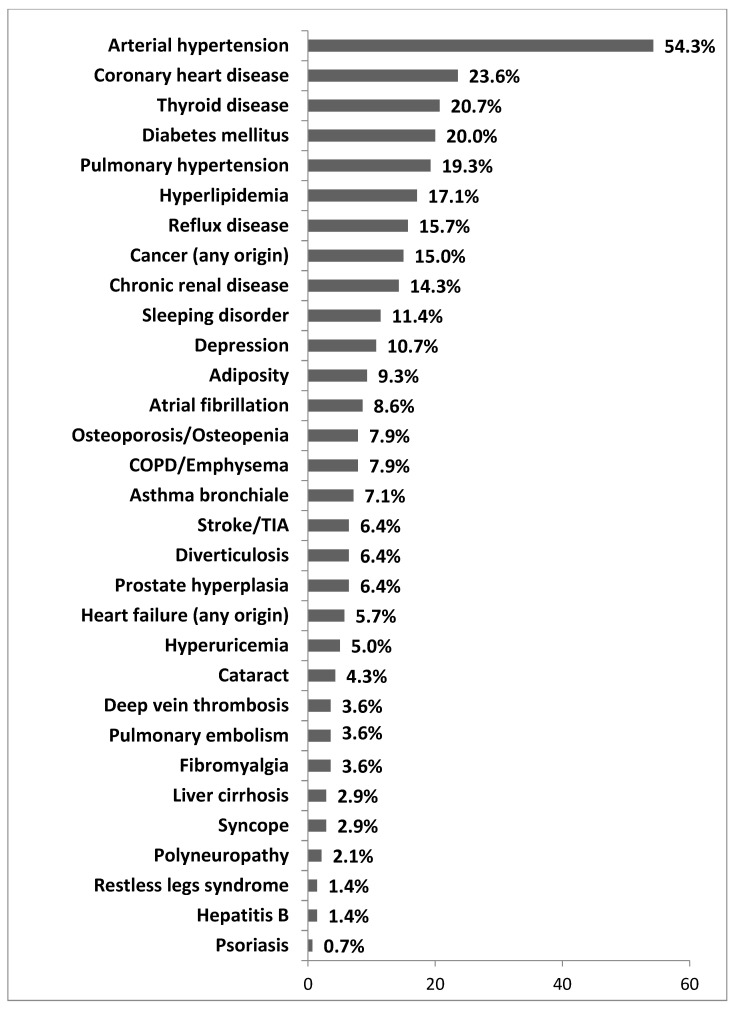
Spectrum of comorbidities in the uILD cohort. Data are given as percentage of all patients. Multiple comorbidities could be reported. Abbreviations: COPD—Chronic obstructive pulmonary disease, TIA—Transient ischemic attack.

Comorbidities were common in patients with uILD and were assessed via both physician and patient questionnaires. As shown in Figure 1, the most common comorbidity in our cohort was arterial hypertension, followed by coronary heart disease, thyroid disease, and diabetes mellitus. A diagnosis of pulmonary hypertension based on right-heart catheterization (RHC) was made in 19.3% of the patients, and this diagnosis was not found to result in a significantly worsened outcome.

**Figure 2:**
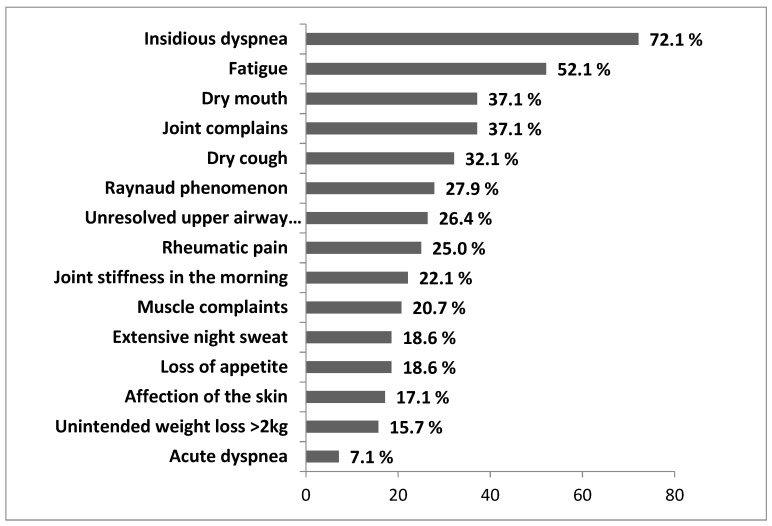
Distribution of self-reported symptoms of uILD patients. Data are presented as percentage of all uILD patients with reported symptoms. Multiple symptoms could be reported.

The most common clinical symptoms self-reported by uILD patients are shown in Figure 2 and included insidious dyspnea (72.1%), fatigue (52.1%), and dry mouth, together with joint complaints (both 37.1%). “Affection of the skin” included erythematous rash and/or other cutaneous affections; “joint complaints” could be of any origin. The term “rheumatic pain” referred to the various painful conditions of rheumatic origin, affecting joints, bones, ligaments, and muscles.

**Figure 3:**
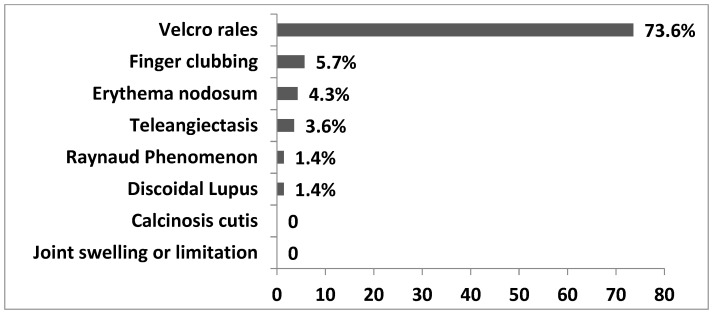
Observed symptoms in the clinical examination of uILD Patients. Data are presented as percentage of all uILD patients with reported symptoms. Multiple symptoms could be reported.

During the physical examination, we found out that velcro rales (73.6%) and finger clubbing (5.7%) were the most common findings, but these were clearly less frequent as compared to our IPF cohort [5]. Additionally, the patients presented with findings such as erythema nodosum (4.3%), teleangiectasis (3.6%), and Raynaud’s phenomenon (1.4%). IPAF criteria were met in 6 (4.3%) patients of the total uILD population. Interestingly, there was a discrepancy between the percentages of patients and physicians reporting Raynaud phenomenon in the same cohort of patients (27.9% and 1.4%, respectively). Figure 3 displays the data.

The severity of dyspnea and impairment of physical activity were evaluated in analogy to the New York Heart Association (NYHA) functional classification at the time point of inclusion into the eurIPFreg. Our cohort showed the following distribution of severity of dyspnea: Grades I–IV 6.4%, 37.1%, 27.9%, and 9.3%, respectively. The data are shown in Figure S1.

### 3.3. Diagnosis of uILD

The reasons for a formal uILD diagnosis were reviewed. We found conflicting clinical, radiological, and pathological data in 46.4% of the cases; patients were too old or frail for lung biopsy in 19.3% of the cases. The tissue sample was insufficient to make a definitive diagnosis in 15.7% of the cases (all transbronchial forceps biopsy) done prior to the era of cryobiopsy, or the biopsy was declined by patients in 12.9% of the cases. In 5.7% of cases, the disease was mild or stable without signs of progression, so there was no benefit for the patient to undergo a lung biopsy. The results are presented in Figure S2.

### 3.4. Pulmonary Function Test (PFT) Data at Baseline

The results of lung function, gas exchange and exercise data are shown in Table 2. As compared to published studies in IPF, our uILD cohort was compatibly sick [5].

### 3.5. HRCT in uILD Patients

In 139 out of 140 uILD patients, an HRCT was performed. Of these, 26.4% showed a definite UIP pattern according to the previous ATS/ERS guideline, 19.3% showed a possible UIP pattern, and 54.3% of patients showed a pattern inconsistent with UIP [24]. Reticular abnormalities were seen in 75.7%, honeycombing with or without traction bronchiectasis in 44.3%, micronodules in 7.1%, and ground-glass opacities in 28.6% of all cases.

**Figure 4:**
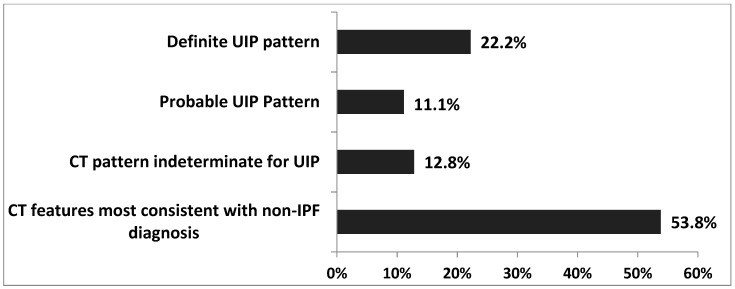
Diagnostic categories of UIP based on the Fleischner Society criteria [26]. Abbreviations: UIP—Usual interstitial pneumonia, CT—Computed tomography, IPF—Idiopathic pulmonary fibrosis.

When applying the UIP CT criteria set up by the Fleischner Society, 22.2% of the patients showed a typical UIP pattern, 11.1% showed a probable UIP pattern, 12.8% showed an indeterminate UIP pattern, and 53.8% showed CT features that were most consistent with a non-IPF diagnosis, as shown in Figure 4 [26].

### 3.6. Bronchoscopy, Bronchoalveolar Lavage (BAL), and Lung Biopsy in uILD

A bronchoscopy was performed in 114 cases (81.43%), and a BAL was performed in 104 cases (74.29%). The BAL differential revealed elevated neutrophil (13.5 ± 16.8%, mean value ± SD), eosinophil (3.7 ± 4.2%), and lymphocyte (17 ± 16.3%) counts, and reduced macrophage counts (66.2 ± 20.6% of all cells, respectively). A lung biopsy was performed in 105 (75%) patients; 71.4% of the biopsies were done via forceps and 7.1% were done via cryobiopsy during bronchoscopy. In 19.4%, a video-assisted thoracic surgery (VATS) was performed, and in 2.0% of the cases, open lung biopsies were conducted.

### 3.7. Impairment of HRQoL in uILD Patients

HRQoL was measured at baseline using the Short Form Health Survey (SF-36). Patients with uILD presented with impairments in physical and mental health, as shown in Table 3.

### 3.8. Treatment for uILD

**Figure 5:**
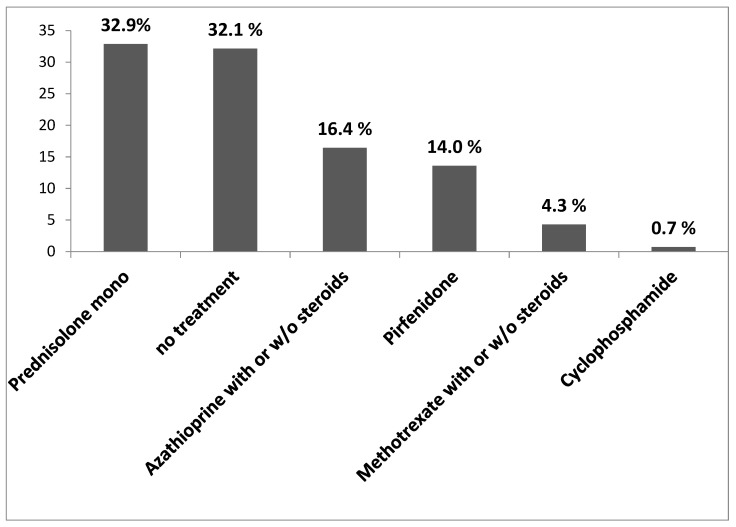
Treatment in uILD cohort. The data are presented as percentage of all uILD patients.

In case of uILD, there is no standardized pharmacological treatment available. Therefore, therapeutic regimes are applied on an individual basis. Our results showed that 32.9% of uILD patients received a mono steroid treatment (prednisolone). In 14% of the cases, the patients received pirfenidone. Figure 5 displays all treatment modalities undertaken in our study cohort as a percentage of all uILD patients.

Of all 75 patients (53.6% of the whole cohort) receiving prednisolone, 19 patients showed a typical UIP pattern, 7 showed a probable UIP pattern, 8 patients showed an indeterminate UIP pattern, and 31 patients showed CT features most consistent with a non-IPF diagnosis (according to the Fleischner Society criteria).

### 3.9. Risk Factors for PFT Decline and Outcome Analyses

In the total cohort, the mean survival time was 57.8 months ± 5.4 SD. The following risk factors were statistically significant for survival: smoking (*p* = 0.033), FVC % pred. at baseline (*p* = 0.008), annual FVC decline ≥10% p.a. (*p* < 0.001), and diffusing capacity of the lung for carbon monoxide (DLco) ≤55% pred. at baseline (*p* < 0.0001).

**Figure 6:**
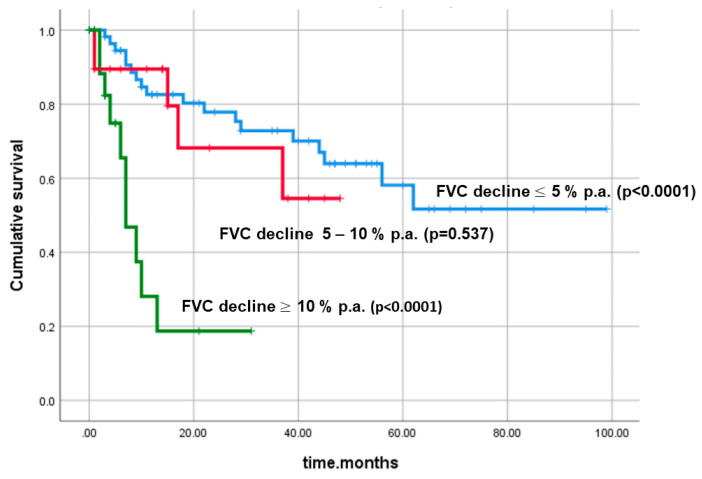
Cumulative survival with regard to forced vital capacity (FVC) decline p.a. (*p* < 0.0001). Abbreviations: FVC—forced vital capacity, % pred.—percentage of predicted value.

Based on the individual FVC decline (% relative per year), the uILD cohort was divided into three main subgroups (≥10%, 5–10%, ≤5%, respectively). Especially patients with an annual FVC decline ≥10% p.a. showed a significantly worse survival, as shown in Figure 6. Furthermore, by Cox regression analysis, we investigated if adding further variables to FVC decline would also play a role in survival (so-called survival function analysis with covariates). Pack years (*p* = 0.968), age at baseline (*p* = 0.574), gender (*p* = 0.829), GAP score at baseline (*p* = 0.339), and velcro rales (*p* = 0.117) did not show statistically significant *p*-values in this analysis. However, adding DLco % pred. to the analysis led to a significant result (*p* < 0.0001).

**Figure 7:**
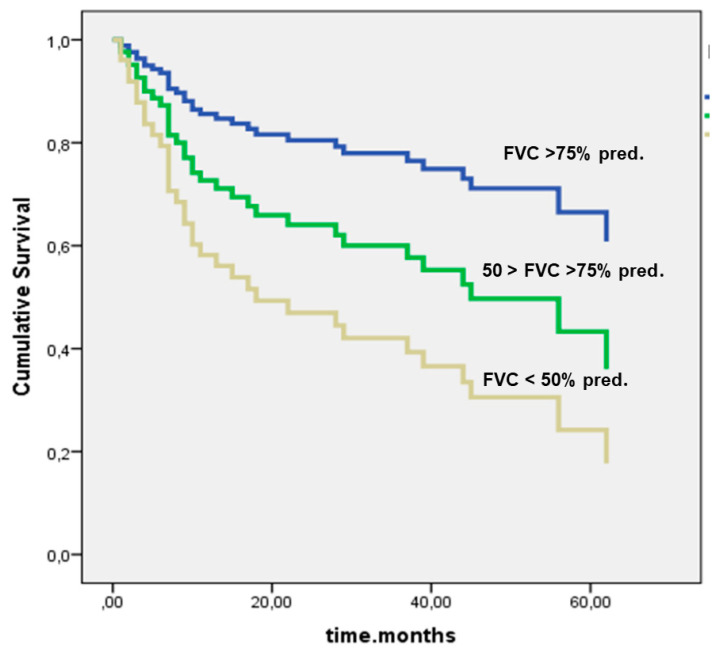
Cumulative survival in dependency of FVC at baseline (*p* = 0.008). Given are Kaplan–Meier curves for cumulative survival, based on definite outcome data. Abbreviations: FVC—forced vital capacity, % pred.—percentage of predicted value.

Figure 7 shows the cumulative survival in dependency of FVC % pred. at baseline (*p* = 0.008). The patients with FVC values under 50% pred. at baseline showed the worst survival (*p* = 0.017).

The outcome analysis in regard to smoking status did reveal a significant impact of this parameter on survival in uILD (*p* = 0.008), as shown in Figure S3. A lower DLco % pred. at baseline was an independent predictor of mortality (*p* < 0.0001 for DLco ≤55% pred. at baseline), as shown in Figure S4.

The GAP stage at baseline did not show a statistically significant impact on survival (*p* = 0.283). We also did not observe any influence on survival with regard to gender (*p* = 0.064), age at baseline (*p* = 0.691), BMI (*p* = 0.352), performance of a lung biopsy (*p* = 0.581), or UIP pattern on HRCT (*p* = 0.162). The 6MWD under 300 m of walking distance had a significant impact on survival (*p* < 0.0001), as shown in Figure S5. In our analysis, prednisolone did not show a significant additional negative impact on survival (*p* = 0.094); the data are presented in Figure S6. The UIP pattern (Fleischner Society Criteria) did not show a statistic significant impact on survival as well (*p* = 0.604, Figure S7).

## 4. Discussion

In this retrospective eurIPFreg analysis, we comprehensively analyzed the clinical features, progression factors, and outcomes of uILD patients. In a Cox regression analysis for survival, we could show that smoking, FVC % pred. at baseline, an FVC decline ≥10% p.a., and a DLco ≤55% pred. at baseline are significantly associated with progressive disease and fatal outcome.

UILD has been the topic of some previous publications. The inconsistent terminology and definitions across studies resulted in different prevalence data and reflect the absence of established diagnostic criteria [26]. In our study, the uILD cohort represented approximately 15% of the total ILD cohort, and approximately 26% of the IIP cohort, similar to the study by Atienza-Mateo et al. [27]. The study by Ryerson et al. reported a percentage of uILD of 10% of the ILD cohort [28]. In the study by Ryerson et al., uILD patients had a comparable mean age (68 years), smoking history (63.6% current/former smokers), or long-term oxygen treatment (LTOT) usage (21.9%) [28]. Although 75% of uILD patients underwent some kind of lung biopsy, still, the diagnosis could not be established, mostly due to conflicting clinical, radiological, and pathological data (in 46.43% of the cases).

In 16% of cases, uILD was diagnosed due to an insufficient amount of lung tissue in the biopsy, and this was exclusively observed in patients undergoing transbronchial forceps biopsies, but in none of the patients receiving cryobiopsy. Cryobiopsies represent an important adjunct for the diagnosis of ILD, enhancing the diagnostic confidence of the treating clinicians in the absence of VATS [29]. In our uILD cohort, recruited between 2009 and 2019, seven patients underwent a cryobiopsy. Meanwhile, this minimally invasive technique has become our new standard for most ILD cases and has almost completely replaced transbronchial forceps biopsy [5].

HRQoL measurements in our uILD cohort were assessed at baseline employing the SF-36. The reported mean value of HRQoL in our cohort forwarded a relevant impairment of both the psychical and the mental health (47.96 out of 100). Both the emotional well-being and general health were reduced (61.91% and 40.16%, respectively). The extent of HRQoL impairment is similar to that in an IPF cohort [7]. To the best of our knowledge, this is the first evaluation of HRQoL in uILD patients.

The accurate diagnosis of ILD is necessary to define the most appropriate management strategy and to predict prognosis. An MDT approach has been considered the gold standard in ILD diagnosis, showing to increase diagnostic accuracy and confidence [30]. All our patients in the uILD cohort were discussed in a MDT board, and also, the entire uILD cohort has been subject of periodical reassessment of uILD diagnosis.

UILD is a diverse collective of patients who frequently have features resembling CTD-ILD or chronic HP [31]. The conception of a provisional diagnosis has been suggested as a useful approach in deciding upon the most appropriate management of ILD patients, especially the ones with fibrosing phenotype [32]. Since there is no standard pharmacological treatment available, depending on the relative likelihood of different diagnoses in a patient with uLD, either antifibrotic or immunosuppressive therapy may be offered on an individual basis [3]. In 55% of our uILD patients, mono steroid or combined immunosuppressive therapy was offered based on the concept of an underlying inflammatory condition. In 14% of the cases, the patients in our study received pirfenidone, as part of the frame of randomized clinical trials (RCT). Drawn against the data of the recently published uILD study, it is to be expected that antifibrotic therapy may become an important therapeutic principle in patients with progressive fibrotic disease [33].

The heterogeneity with regard to the prevalence of uILD reported by different study groups is most likely due to differences in definitions of uILD, different thresholds for assigning diagnoses, as well as variability in understanding clinical, radiological, and pathological features [34]. There are patients with ILD who remain unclassifiable despite a surgical lung biopsy and patients who are unclassifiable in the absence of a lung biopsy [32]. The inability to establish a clear diagnosis may result in delayed treatment in uILD patients with variable clinical courses and prognoses, who potentially might benefit from antifibrotic therapy.

Due to the heterogeneity of uILD, it is also essential to identify reliable prognostic factors indicating the risk of PFT decline and outcome, together with treatment response in a comprehensive non-selected patient’s cohort. The Fleischner Society criteria for the radiographic diagnosis of UIP are helpful in assigning a diagnosis of IPF, but at the same time, we recognize and specify different degrees of confidence in the diagnosis [35]. By applying these diagnostic criteria to our uILD cohort, we showed that in 53.84% of uILD cases, the CT features were most consistent with a non-IPF diagnosis.

The mean survival time in our cohort was 57.81 months, i.e., comparable to survival in IPF. Guler et al. reported survival rates in uILD of 84% to 89% (1 year), 70% to 76% (2 years), and 46% to 70% (5 years), respectively. Unfortunately, we did not observe any influence on survival of gender (*p* = 0.064), age at baseline (*p* = 0.536), GAP score at baseline (*p* = 0.303), or performance of lung biopsy (*p* = 0.581). However, we could show that baseline FVC, Dlco, and 6MWD and, even more important, annual FVC decline had a deep impact on outcome. Such observation strengthens the “lumping” approach in ILDs, according to which the disease behavior, more than a formal diagnosis, may help to identify a group of patients at risk of progression and death [36]. This opportunity may allow pulmonologists to move toward antifibrotic therapy in progressive uILD.

## 5. Study Limitations

The interpretation of the real-world data has several limitations. First, as a databank, the information available for each patient is based on clinical practice instead on a trial protocol. Additionally, the co-medication, as well as comorbidities, are partly self-reported as a fragment of the structured patient questionnaire and thus might lack accuracy.

## 6. Conclusions

Similar to IPF, the most important known prognostic determinants for mortality in uILD are decline in lung function and smoking. The use of Fleischner diagnostic criteria might allow further differentiation and prognostic evaluation together with new therapeutic options for patients, who currently have no established approach to therapy. The indicators for uILD patients for treatment response to antifibrotics have to be established in the future.

## Figures and Tables

**Table 1:** Clinical characteristic of the unclassifiable interstitial lung diseases (uILD) cohort.

Demographic Parameters	Values
Male (*n*, %)	81, 57.9%
BMI (mean value + SD (kg-m²)),	24.2 ± 4.8
range (minimum- maximum, kg-m²)	16–32
Age at enrollment into the registry mean value + SD (years)	68.2 ± 11.0
Time between onset of symptoms and inclusion into eurIPFreg (mean value ± SD (months))	6.7 ± 12.6
Smokers (yes, no, missing data in %)	71%, 54%, 15%
Kind of smoker (current, former, never smoked, missing data, %)	7.1%, 43.6%, 38.6%, 10.7%
Number of pack-years (mean value ± SD)	28.5 ± 20.2
GAP Stage I /II /III (*n*, %)	43, 30.1%/45, 31.5%/18, 12.6%
Velcro rales at auscultation (*n*, %)	103, 73.6%

Abbreviations: BMI—Body mass index, eurIPFreg—European idiopathic pulmonary fibrosis Registry, SD—Standard deviation, GAP—Gender, Age, Physiology, *n*—Number.

**Table 2:** Results of lung function and gas exchange data in the uILD cohort.

Parameters	Mean Value ± SD
VC (% pred.)	69.3 ± 21.3
FVC (% pred.)	67.8 ± 22.1
FEV 1 (% pred.)	76.3 ± 55.6
FEV 1% FVC (% pred.)	106.0 ± 16.8
RV (% pred.)	94.3 ± 60.8
TLC (% pred.)	74.6 ± 22.7
DLco (% pred.)	43.8 ± 26.3
pO2 (mm Hg) at rest	70.7 ± 12.6
pCO2 (mm Hg) at rest	38.5 ± 4.6
6 MWD (m)	367.8 ± 121.9
Patients receiving LTOT (*n*;%)	41; 29.3
Oxygen flow at rest (l/min)	3.0 ± 1.8
Oxygen flow at exertion (l/min)	4.2 ± 2.5

**Abbreviations:** VC—Vital capacity, FVC—Forced vital capacity, FEV1—Forced expiratory volume; RV—Residual volume, TLC—Total lung capacity, DLco—Diffusing capacity of the lung for carbon monoxide, pO2—Partial pressure of oxygen, pCO2—Partial pressure of carbon dioxide, 6MWD—Six-minute walking distance, LTOT—Long-term oxygen treatment, m—Meters, min—Minutes.

**Table 3:** Results of health-related quality of life (HRQoL) analysis in uILD cohort.

HRQoL Variables	Mean Value	Min	Maximum	SD
Physical functioning %	39.5	0	100	29.1
Role limitations due to physical health %	33.5	0	100	42.0
Role limitations due to emotional problems %	62.0	0	100	46.4
Energy/fatigue	40.6	0	87.0	21.6
Emotional well-being %	61.9	0	100	21.2
Social functioning %	60.1	0	100	30.9
Pain %	62.9	0	100	33.1
General health %	40.2	0	85	18.7
Health change %	30.3	0	100	23.5
SF 36 mean value	48.0	2.78	88.2	21.1

**Abbreviations:** SF 36—Short Form Health Survey. Score for each domain range from 0 to 100, with a higher score defining a more favorable health state, SD—standard deviation.

## Data Availability

The datasets used and analyzed during the current study are available from the corresponding author on reasonable request.

## Supplementary Materials

The following are available online at https://www.mdpi.com/2077-0383/9/8/2499/s1, Figure S1: Severity of dyspnea and impairment of physical activity (NYHA I-IV) in uILD cohort at baseline. The data are presented as percentage of all uILD patients. Abbreviations: NYHA- New York Heart Association, Figure S2: Underlying reasons for the diagnosis of uILD. Data are presented as percentage of all uILD patients, Figure S3: Cumulative survival and smoking status (*p* = 0.008), Figure S4: Cumulative survival and DLco % pred. at baseline (*p* < 0.0001). Abbreviations: DLCO- diffusing capacity of the lung for carbon monoxide, Figure S5: Cumulative survival and 6MWD at baseline (*p* < 0.0001). Abbreviations: 6MWD- six minutes walking distance, m-meters, Figure S6: Impact of prednisolone on survival in uILD (*p* = 0.094), Figure S7: Impact of UIP pattern in High-resolution CT (Fleischner Society Criteria) on survival in uILD (p=0.604). Abbreviations: UIP- usual interstitial pneumonia, CT- Computed Tomography, IPF- idiopathic pulmonary fibrosis.

## Trial registration

The eurIPFreg and eurIPFbank are listed in ClinicalTrials.gov (NCT02951416).

## Copyright and license agreement

All authors of the manuscript have read and agreed to its content and are accountable for all aspects of the accuracy and integrity of the manuscript in accordance with journal criteria.

## References

[1] Antoniou K.M., Margaritopoulos G.A., Tomassetti S., Bonella F., Costabel U., Poletti V. (2014). Interstitial lung disease. Eur. Respir. Rev..

[2] Cottin V., Hirani N.A., Hotchkin D.L., Nambiar A.M., Ogura T., Otaola M., Skowasch D., Park J.S., Poonyagariyagorn H.K., Wuyts W. (2018). Presentation, diagnosis and clinical course of the spectrum of progressive-fibrosing interstitial lung diseases. Eur. Respir. Rev..

[3] Wong A.W., Ryerson C.J., Guler S.A. (2020). Progression of fibrosing interstitial lung disease. Respir. Res..

[4] Barratt S.L., Creamer A., Hayton C., Chaudhuri N. (2018). Idiopathic Pulmonary Fibrosis (IPF): An Overview. J. Clin. Med..

[5] Guenther A., Krauss E., Tello S., Wagner J., Paul B., Kuhn S., Maurer O., Heinemann S., Costabel U., Barbero M.A.N. (2018). The European IPF registry (eurIPFreg): baseline characteristics and survival of patients with idiopathic pulmonary fibrosis. Respir. Res..

[6] Loeh B., Brylski L.T., von der Beck D., Seeger W., Krauss E., Bonniaud P., Crestani B., Vancheri C., Wells A.U., Markart P. (2019). Lung CT Densitometry in Idiopathic Pulmonary Fibrosis for the Prediction of Natural Course, Severity, and Mortality. Chest.

[7] Witt S., Krauss E., Barbero M.A.N., Müller V., Bonniaud P., Vancheri C., Wells A.U., Vasakova M., Pesci A., Klepetko W. (2019). Psychometric properties and minimal important differences of SF-36 in Idiopathic Pulmonary Fibrosis. Respir. Res..

[8] Neurohr C., Behr J. (2015). Changes in the current classification of IIP: A critical review. Respirology.

[9] Oliveira D.S., Araújo Filho J.d.A., Paiva A.F.L., Ikari E.S., Chate R.C., Nomura C.H. (2018). Idiopathic interstitial pneumonias: review of the latest American Thoracic Society/European Respiratory Society classification. Radiol. Bras..

[10] Aburto M., Herráez I., Iturbe D., Jiménez-Romero A. (2018). Diagnosis of Idiopathic Pulmonary Fibrosis: Differential Diagnosis. Med. Sci. (Basel).

[11] Hyldgaard C., Bendstrup E., Wells A.U., Hilberg O. (2017). Unclassifiable interstitial lung diseases: Clinical characteristics and survival. Respirology.

[12] Brown A.W. (2018). Unclassifiable Interstitial Lung Disease: Time to Shrink the Black Box. Ann. Am. Thorac. Soc..

[13] Guler S.A., Ryerson C.J. (2018). Unclassifiable interstitial lung disease: from phenotyping to possible treatments. Curr. Opin. Pulm. Med..

[14] Leung S.C., Churg A.M., Leipsic J.A., Levy R.D., Wilcox P.G., Ryerson C.J. (2015). Unclassifiable interstitial lung disease: an unresolved diagnostic dilemma. Respirol. Case Rep..

[15] Jones K.D. (2018). Unclassifiable interstitial lung disease: a pathologist’s perspective. Eur. Respir. Rev..

[16] Nakamura Y., Sugino K., Kitani M., Hebisawa A., Tochigi N., Homma S. (2018). Clinico-radio-pathological characteristics of unclassifiable idiopathic interstitial pneumonias. Respir. Investig..

[17] Luppi F., Wells A.U. (2016). Interstitial pneumonitis with autoimmune features (IPAF): a work in progress. Eur. Respir. J..

[18] Collins B.F., Spiekerman C.F., Shaw M.A., Ho L.A., Hayes J., Spada C.A., Stamato C.M., Raghu G. (2017). Idiopathic Interstitial Pneumonia Associated With Autoantibodies: A Large Case Series Followed Over 1 Year. Chest.

[19] Fischer A., Antoniou K.M., Brown K.K., Cadranel J., Corte T.J., Du Bois R.M., Lee J.S., Leslie K.O., Lynch D.A., Matteson E.L. (2015). An official European Respiratory Society/American Thoracic Society research statement: interstitial pneumonia with autoimmune features. Eur. Respir. J..

[20] Ryerson C.J., Vittinghoff E., Ley B., Lee J.S., Mooney J.J., Jones K.D., Elicker B.M., Wolters P.J., Koth L.L., King T.E. (2014). Predicting survival across chronic interstitial lung disease: the ILD-GAP model. Chest.

[21] Raghu G., Rochwerg B., Zhang Y., Garcia C.A.C., Azuma A., Behr J., Brozek J.L., Collard H.R., Cunningham W., Homma S. (2015). An Official ATS/ERS/JRS/ALAT Clinical Practice Guideline: Treatment of Idiopathic Pulmonary Fibrosis. An Update of the 2011 Clinical Practice Guideline. Am. J. Respir. Crit. Care Med..

[22] Raghu G., Remy-Jardin M., Myers J.L., Richeldi L., Ryerson C.J., Lederer D.J., Behr J., Cottin V., Danoff S.K., Morell F. (2018). Diagnosis of Idiopathic Pulmonary Fibrosis. An Official ATS/ERS/JRS/ALAT Clinical Practice Guideline. Am. J. Respir. Crit. Care Med..

[23] Travis W.D., Costabel U., Hansell D.M., King T.E., Lynch D.A., Nicholson A.G., Ryerson C.J., Ryu J.H., Selman M., Wells A.U. (2013). An official American Thoracic Society/European Respiratory Society statement: Update of the international multidisciplinary classification of the idiopathic interstitial pneumonias. Am. J. Respir. Crit. Care Med..

[24] Raghu G., Collard H.R., Egan J.J., Martinez F.J., Behr J., Brown K.K., Colby T.V., Cordier J.-F., Flaherty K.R., Lasky J.A. (2011). An official ATS/ERS/JRS/ALAT statement: idiopathic pulmonary fibrosis: evidence-based guidelines for diagnosis and management. Am. J. Respir. Crit. Care Med..

[25] Mavroudi M., Papakosta D., Kontakiotis T., Domvri K., Kalamaras G., Zarogoulidou V., Zarogoulidis P., Latka P., Huang H., Hohenforst-Schmidt W. (2018). Sleep disorders and health-related quality of life in patients with interstitial lung disease. Sleep Breath..

[26] Traila D., Oancea C., Tudorache E., Mladinescu O.F., Timar B., Tudorache V. (2018). Clinical profile of unclassifiable interstitial lung disease: Comparison with chronic fibrosing idiopathic interstitial pneumonias. J. Int. Med. Res..

[27] Atienza-Mateo B., Remuzgo-Martínez S., Mora Cuesta V.M., Iturbe-Fernández D., Fernández-Rozas S., Prieto-Peña D., Calderón-Goercke M., Corrales A., Blanco Rodríguez G.B., Gómez-Román J.J. (2020). The Spectrum of Interstitial Lung Disease Associated with Autoimmune Diseases: Data of a 3.6-Year Prospective Study from a Referral Center of Interstitial Lung Disease and Lung Transplantation. J. Clin. Med..

[28] Ryerson C.J., Urbania T.H., Richeldi L., Mooney J.J., Lee J.S., Jones K.D., Elicker B.M., Koth L.L., King T.E., Wolters P.J. (2013). Prevalence and prognosis of unclassifiable interstitial lung disease. Eur. Respir. J..

[29] Troy L.K., Hetzel J. (2020). Lung cryobiopsy and interstitial lung disease: What is its role in the era of multidisciplinary meetings and antifibrotics?. Respirology.

[30] Chaudhuri N., Spencer L., Greaves M., Bishop P., Chaturvedi A., Leonard C. (2016). A Review of the Multidisciplinary Diagnosis of Interstitial Lung Diseases: A Retrospective Analysis in a Single UK Specialist Centre. J. Clin. Med..

[31] Skolnik K., Ryerson C.J. (2016). Unclassifiable interstitial lung disease: A review. Respirology.

[32] Ryerson C.J., Corte T.J., Lee J.S., Richeldi L., Walsh S.L.F., Myers J.L., Behr J., Cottin V., Danoff S.K., Flaherty K.R. (2017). A Standardized Diagnostic Ontology for Fibrotic Interstitial Lung Disease. An International Working Group Perspective. Am. J. Respir. Crit. Care Med..

[33] Behr J., Neuser P., Prasse A., Kreuter M., Rabe K., Schade-Brittinger C., Wagner J., Günther A. (2017). Exploring efficacy and safety of oral Pirfenidone for progressive, non-IPF lung fibrosis (RELIEF) - a randomized, double-blind, placebo-controlled, parallel group, multi-center, phase II trial. BMC Pulm. Med..

[34] Guler S.A., Ellison K., Algamdi M., Collard H.R., Ryerson C.J. (2018). Heterogeneity in Unclassifiable Interstitial Lung Disease. A Systematic Review and Meta-Analysis. Ann. Am. Thorac. Soc..

[35] Lynch D.A., Sverzellati N., Travis W.D., Brown K.K., Colby T.V., Galvin J.R., Goldin J.G., Hansell D.M., Inoue Y., Johkoh T. (2018). Diagnostic criteria for idiopathic pulmonary fibrosis: a Fleischner Society White Paper. Lancet Respir. Med..

[36] de Sadeleer L.J., Goos T., Yserbyt J., Wuyts W.A. (2020). Towards the Essence of Progressiveness: Bringing Progressive Fibrosing Interstitial Lung Disease (PF-ILD) to the Next Stage. J. Clin. Med..

