# Supplementary material for: Clinical and Functional Characteristics of Patients with Unclassifiable Interstitial Lung Disease (uILD): Long-Term Follow-Up Data from European IPF Registry (eurIPFreg)
Source: J Clin Med. 2020 Aug 3;9(8):2499. doi: 10.3390/jcm9082499 (PMC7464480; doi:10.3390/jcm9082499)
Supplement: Supplementary file 1 [file jcm-09-02499-s001.pdf]

Supplementary figures:

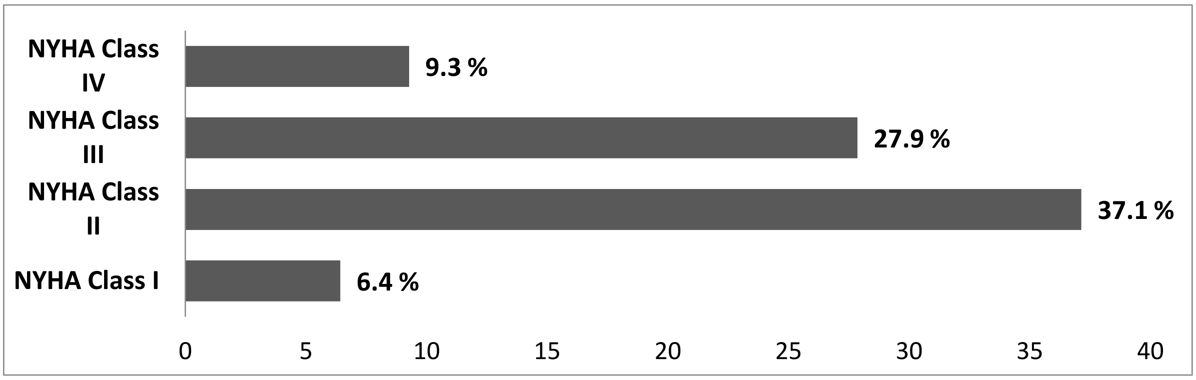

Supplementary Figure 1. Severity of dyspnea and impairment of physical activity (NYHA I-IV) in uILD cohort at baseline. The data are presented as percentage of all uILD patients. Abbreviations: NYHA- New York Heart Association.

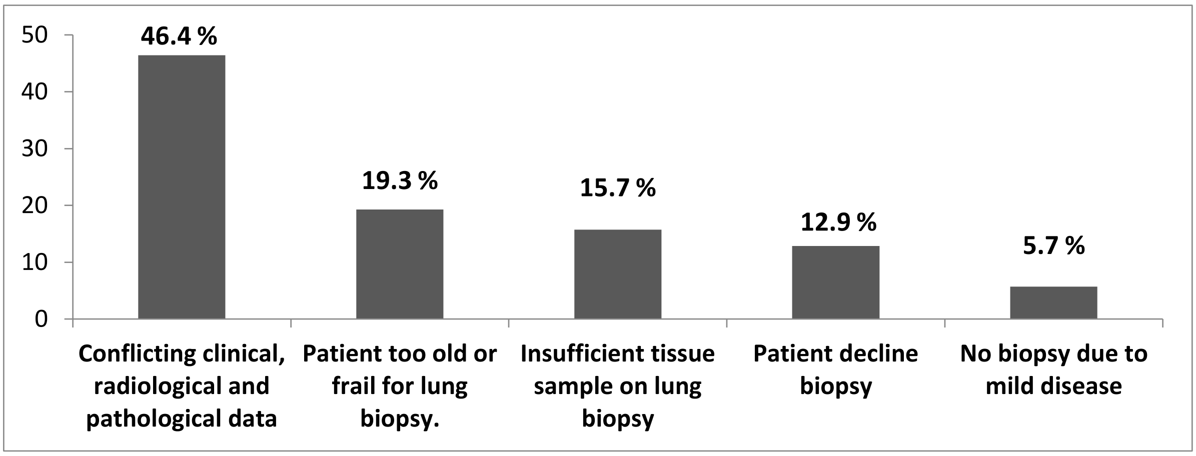

Supplementary Figure 2. Underlying reasons for the diagnosis of uILD. Data are presented as percentage of all uILD patients.

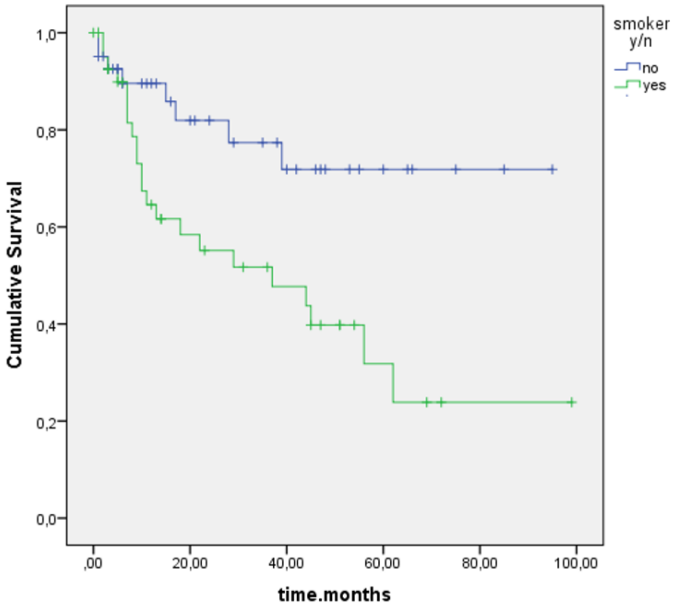

Supplementary Figure 3. Cumulative survival and smoking status (p=0.008)

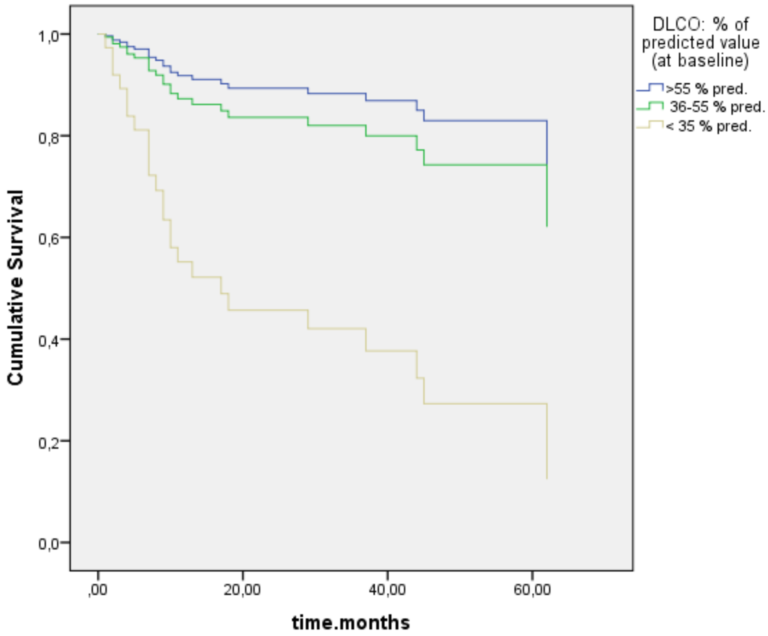

Supplementary Figure 4. Cumulative survival and DLCO % pred. at baseline (p<0.0001).

Abbreviations: DLCO- diffusing capacity of the lung for carbon monoxide.

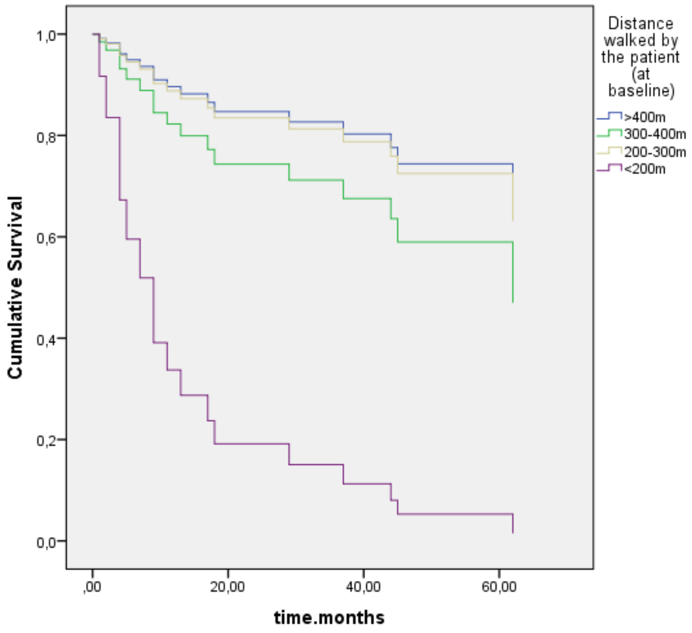

Supplementary Figure 5. Cumulative survival and 6MWD at baseline (p<0.0001). Abbreviations: 6MWD- six minutes walking distance, m-meters.

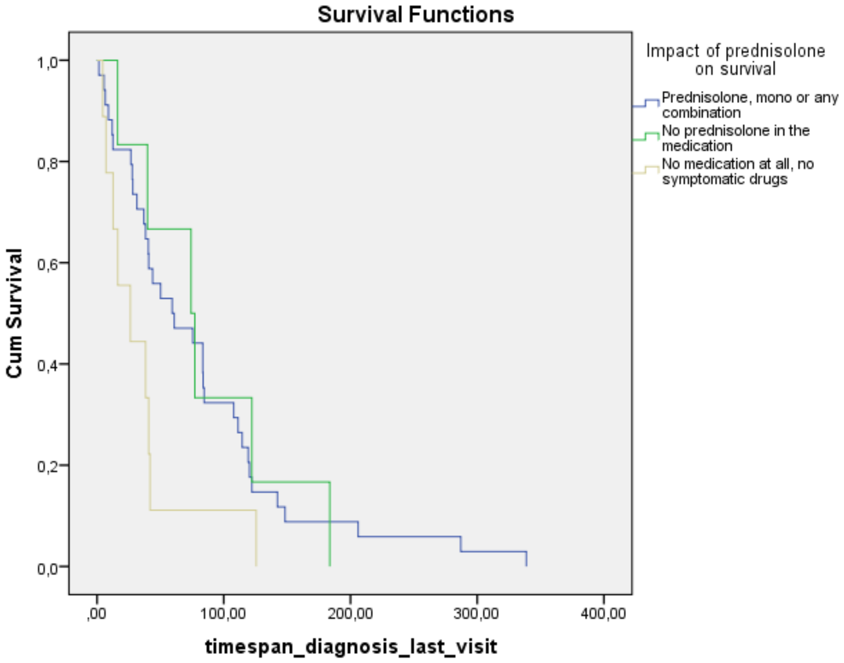

Supplementary Figure 6. Impact of prednisolone on survival in uILD (p=0.094).

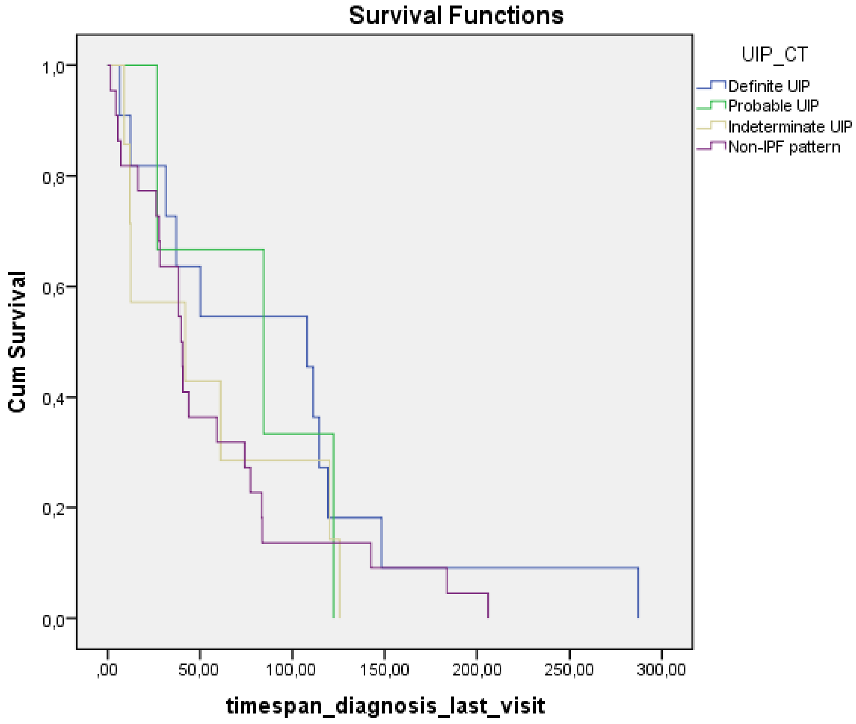

Supplementary Figure 7. Impact of UIP pattern in High-resolution CT (Fleischner Society Criteria) on survival in uILD (p=0.604). Abbreviations: UIP- usual interstitial pneumonia, CT- Computed Tomography, IPF- idiopathic pulmonary fibrosis.
